# Trastuzumab and pertuzumab in circulating tumor DNA ERBB2-amplified HER2-positive refractory cholangiocarcinoma

**DOI:** 10.1038/s41698-019-0091-4

**Published:** 2019-08-19

**Authors:** Bhavya Yarlagadda, Vaishnavi Kamatham, Ashton Ritter, Faisal Shahjehan, Pashtoon M. Kasi

**Affiliations:** 1 Division of Oncology, CORPS Program, Department of Internal Medicine, Mayo Clinic, Jacksonville, FL USA; 2 Division of Hematology, Oncology and Blood & Bone Marrow Transplantation, Department of Internal Medicine, University of Iowa, Iowa City, IA USA

**Keywords:** Targeted therapies, Biliary tract cancer, Cancer genetics, Tumour heterogeneity, Tumour biomarkers

## Abstract

Cholangiocarcinoma is a heterogeneous and target-rich disease with differences in actionable targets. Intrahepatic and extrahepatic types of cholangiocarcinoma differ significantly in clinical presentation and underlying genetic aberrations. Research has shown that extrahepatic cholangiocarcinoma is more likely to be associated with *ERBB2* (HER2) genetic aberrations. Various anti-HER2 clinical trials, case reports and other molecular studies show that HER2 is a real target in cholangiocarcinoma; however, anti-HER2 agents are still not approved for routine administration. Here, we show in a metastatic cholangiocarcinoma with *ERBB2* amplification identified on liquid biopsy (circulating tumor DNA (ctDNA) testing), a dramatic response to now over 12 months of dual-anti-HER2 therapy. We also summarize the current literature on anti-HER2 therapy for cholangiocarcinoma. This would likely become another treatment option for this target-rich disease.

## Introduction

Cholangiocarcinoma (CCA) is a lethal tumor arising from the epithelium of the bile ducts that most often presents at an advanced stage. A total of 10–20% of primary hepatic malignancies are CCA, which is the second most common hepatobiliary malignancy.^1^ Risk factors for development of CCA include cigarette smoking, heavy alcohol use, primary sclerosing cholangitis, viral hepatitis B and C, and genetic diseases such as Lynch syndrome.^2^

CCA, including gallbladder cancer, is a heterogeneous group of diseases. Multiple aberrations can be seen, including *ERBB2*, microsatellite instability high (MSI-H), *IDH1*, *BRAF*, *FGFR* fusion, *BRCA*/DNA-repair related, *MET* amplification, *NTRK* fusion, *TP53*, *KRAS*, *ARID1A*, *MCL1*, *PBRM1*, *SMAD4*, *FBXW7*, and *CDKN2A*.^3–10^ As in most cancers, these aberrations are not actionable; however, cholangiocarcinoma is a very “target rich” disease; *ERBB2*, *MSI-H*, *IDH1*, *BRAF*, *FGFR fusion*, *MET amplification*, and *NTRK* fusion^5,6,8,9,11^ may be susceptible to approved or off-label or presence of drugs treatments through clinical trials that are showing promise. Tumor-agnostic FDA-approved immunotherapy for MSI-H tumors and larotrectinib for NTRK-fusion tumors are showing promise.^9,12^ A number of published cases and open clinical trials with early results have demonstrated activity in *IDH1*, *BRAF*-mutant, *MET*-amplified, *ERBB2*-amplified, and *FGFR-* fusion tumors.^6,7,13,14^

The *ERBB2* gene encodes for the protein HER2 or HER2/neu, which is a tyrosine kinase family receptor. Breast, stomach and esophageal cancers have well-established associations with *ERBB2* genetic aberrations; and are approved for anti-HER2.^15,16^ However, reports have also shown the finding of HER2 aberrations in CCA and urinary bladder cancers.^17^ These are interestingly seen more with extrahepatic cholangiocarcinomas and gallbladder cancers as opposed to intrahepatic cholangiocarcinomas.

We present a 71-year-old female diagnosed with metastatic CCA with *ERBB2* (HER2) 3+ amplification determined by circulating tumor DNA (ctDNA) testing (“liquid biopsy”) and confirmed by tissue-based testing. She was treated with dual HER2-directed therapy (trastuzumab/pertuzumab), and responded very well with regression of tumor on imaging as well as normalization of liver function tests and tumor marker levels including improvement in her overall clinical status. Serial ctDNA testing (Table 1) alongside standard of care imaging continues to show ongoing durable control >12 months into therapy. Other case reports and studies highlighting the association of HER2 and CCA are also presented. The patient provided written informed consent to report this case.

**Table 1:** Serial circulating tumor DNA evaluation in our patient

Amplification or mutation	Amplification or %cfDNA	Amplification or %cfDNA	Amplification or %cfDNA	Amplification or %cfDNA	Amplification or %cfDNA	Amplification or %cfDNA
	Jan 2018	Mar 2018	May 2018	Jun 2018	Aug 2018	Oct 2018
Treatment	Gemcitabine + Carboplatin chemotherapy		Dual-anti-HER2 blockade (Pertuzumab + Trastuzumab)			
*ERBB2* (HER2) amplification	+++ Plasma Copy Number: 68.5	+++ Plasma Copy Number: 70.6	+++ Plasma Copy Number: 52.6	+++ Plasma Copy Number: 46.0	+++ Plasma Copy Number: 48.3	+++ Plasma Copy Number: 6.4
*Myc* amplification	++ Plasma Copy Number: 3.3	++ Plasma Copy Number: 2.7	++ Plasma Copy Number: 3.1	++ Plasma Copy Number: 3.3	++ Plasma Copy Number: 2.7	ND^a^
*Tumor markers*						
CA-19-9 (units/ml)	99	462	366	154	90	57
*Clonal aberrations*						
*TP53 Y220C*	ND	60.7%	35.6%	33.0 %	32.9%	2.1%
*Subclonal aberrations*						
TP53 P190S	ND	0.1%	ND	ND	ND	ND
NTRK1 P453P	ND	ND	ND	0.08%	ND	ND
MYC P177P	ND	ND	ND	0.1%	ND	0.2%
PDGFRA E298D	ND	ND	ND	ND	ND	0.2%
FGFR2 S24F	ND	ND	ND	ND	ND	0.1%

*ND* not detectable

## Results

### Case presentation

A 71-year-old female presented in December 2017 after diagnosis of metastatic CCA. Ultrasound demonstrated innumerable liver lesions, which on confirmed on follow-up computed tomography and magnetic resonance imaging showing multiple liver lesions consistent with CCA with intrahepatic metastases. The patient was also noted to have metastatic periportal and aortocaval adenopathy.

**Fig. 1:**
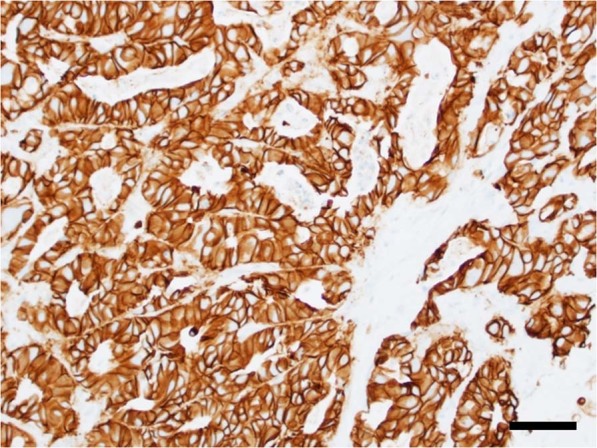
Tumor with intense HER2/Neu 3+ positivity on immunohistochemistry (scale 50 μm)

The patient was not a candidate for surgical intervention due to bilobar disease with innumerable liver lesions. Platinum-based chemotherapy was recommended. She was started on a combination carboplatin and gemcitabine (not cisplatin due to age and sensorineural hearing loss). A baseline ctDNA test was obtained as part of the standard of care at our institution for patients with gastrointestinal malignancies, and in particular cholangiocarcinoma, given the target-rich nature of the disease. Testing is performed through commercially available platforms. Guardant360 testing showed *ERBB2* (HER2) amplification 3+ and was confirmed through tumor tissue-based immunohistochemistry as well as genetic testing through the commercially available platform by Tempus confirming this (Fig. 1). Given the liver-predominant nature of the disease, upfront Y90-radioembolization was also planned. However, within 2 months, the patient had rapid progression of disease with rising tumor markers, rising ctDNA levels, derangement in liver function tests and decline in clinical condition.

We initially considered the patient’s eligibility for the MyPathway Study which has an arm for dual-anti-HER2 therapy.^18^ However, due to her rapid decline was deemed ineligible. Best supportive/palliative care vs. off-label anti-HER2 therapy was discussed. Given the patient’s excellent overall baseline performance status, we began off-label treatment with anti-HER2 pertuzumab/trastuzumab combination therapy.

**Fig. 2:**
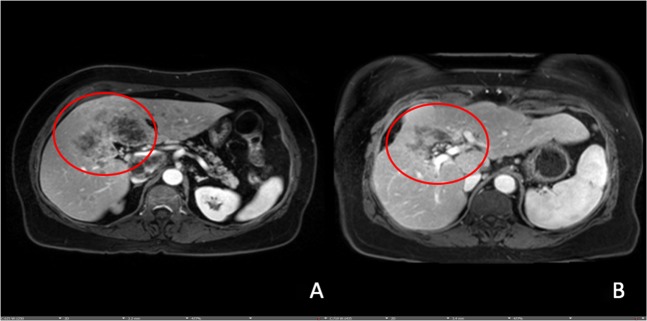
MRI pre- and post-treatment with decreased size and enhancement of lesions in liver

Immediate and rapid improvement of tumor markers was noted. After just one treatment, the patient’s liver function tests improved; most notably, the dominant *TP53* mutation reduced from 60.7 to 2.1% (Table 1). Clinical variables continued to improve rapidly through and continues to do so now over a year into treatment. Scans also showed excellent ongoing durable response (Fig. 2a, b).

## Discussion

CCA is classified into intrahepatic and extrahepatic types based on anatomic location. These are clinically different from each other in terms of presentation, and more importantly the type and frequency of genetic aberrations. Generally, intrahepatic CCA is associated with *IDH1*, *FGFR* fusion and *BRCA*/DNA-repair gene alterations, while extrahepatic CCA is more frequently associated with *ERBB2* genetic aberrations.^19^ A number of these aberrations are potentially actionable in terms of FDA-approved therapies and/or availability of clinical trials or off-label therapies. For a rare disease like CAA, even the latter are pertinent. There are several well-established risk factors related to the development of CCA. However, it has been reported that only 10% of intrahepatic CCA is associated with these risk factors and the remaining 90% are sporadic.^20^

What is unique about our case is that the HER2 aberration was detected on circulating tumor DNA testing (“liquid biopsy”). This was later confirmed on tissue-based testing (Fig. 1). This would not have been known otherwise. Furthermore, for a patient with refractory cholangiocarcinoma that progresses on frontline therapy, prognosis in such situations is in the order of weeks–months. The fact that patient continues to have durable control 12 months and beyond is very encouraging and unprecedented. Progression-free survival of second-line chemotherapeutic regimens is in the order of 4 months on average.

Several studies have described the association of CCA subgroups with various aberrations (Table 2). Churi et al. examined 75 CCA patients for tumoral genetic differences. They identified *TP53*, *KRAS*, *ARID1A*, *IDH1*, *MCL1*, and *PBRM1* mutations in 35%, 24%, 20%, 18%, 16%, and 11% of intrahepatic CCA patients, respectively. *TP53*, *KRAS*, *ERBB2*, *SMAD4*, *FBXW7*, and CDKN2A were found in 45%, 40%, 25%, 25%, 15%, and 15% of extrahepatic CCA patients, respectively.^19^ The finding of a higher proportion of *ERBB2* mutations in extrahepatic CCA and gallbladder cancer suggests that these patients are more likely to carry it as opposed to intrahepatic CCA. Yoshikawa et al. measured HER2/neu expression in 236 cases of CCA by immunohistochemistry. They reported a 0.9% and 8.5% positive HER2/neu expression rate in intrahepatic and extrahepatic CCAs, respectively.^21^ In a study by Lee et al., HER2 and HER3 overexpression was observed in 6% and 39%, respectively, in patients with extrahepatic CCA, with HER3 overexpression associated with worse survival.^22^ A systemic review and meta-analysis by Galdy et al. showed a stronger rate of HER2 expression in extrahepatic CCA (~20%) vs. intrahepatic CCA (<5%).^23^ Summary of the studies showing *HER2* aberrations in CCA and treatment outcomes where available are presented in Table 2.

**Table 2:** HER2/Neu (*ERBB2*) expression in cholangiocarcinoma and response to therapy

Study	*N*/year	Patients	Therapy used	Outcome (response/duration—months)
Javle et al. (MyPathway Clinical trial—NCT02091141)^18^	11 patients/ 2017	Refractory metastatic biliary cancer with HER2 amplification/overexpression (8 patients) or putative activating mutations by gene sequencing (3 patients),^18^ FISH, or IHC	Pertuzumab + trastuzumab until disease progression or unacceptable toxicity	4 patients had partial responses (PR) and 3 had stable disease (SD) for >4 months
Javle et al.^27^	2015	Gallbladder cancer—9 patients CCA—5 patients	Trastuzumab, lapatinib, or pertuzumab	1 CR, 4 PR and 4 SD in gallbladder cases; 1 mixed response on lapatinib with HER2 mutation. Duration of response 8–160+ weeks (median 40 weeks); 3 still on therapy. 1 HER2 amplification was acquired post-FGFR fusion therapy. No responses in CCA cases
Nam et al.^32^	2016	3 patients with gallbladder cancer	All 3 patients received trastuzumab alongside single- or doublet chemotherapy	2 PR; 1 SD. Duration of response 12–34 weeks.
Subbaiah et al.^33^	2013	1 patient with gallbladder cancer	Phase-1 study: anti-HER-2/neu antibody trastuzumab and the HER-2/neu tyrosine kinase inhibitor lapatinib, and then with trastuzumab and erlotinib	SD Duration of response 10.8 months
Churi et al.^19^	2014	2 patients with HER2/neu mutations	Trastuzumab and lapatinib, respectively	No responses observed
Lee et al.^22^	2012	Overexpression of HER2 and HER3 was observed in 6 % (13/224) and 39 % (90/230) of EHCCs, respectively	No treatment data provided	–
Mckay et al.^34^	2011	Expression data only; only CC patients; no gallbladder cancer patients. 19 cases were positive per IHC (10 were scored as +2 and one as +3)	No treatment data provided	–
Sorscher et al.^30^	2013	Gallbladder cancer—1 patient HER2/neu+++	Trastuzumab + weekly paclitaxel (chemo later discontinued)	Marked partial response (5.4 cm → 1.3 cm). Duration of response ongoing (at least 6 months)
Jung et al.^28^	2017	3/84 patients with HER2/neu+ distal extrahepatic CC (3.6%)	No treatment data provided	–
Yoshikawa et al.^21^	2007	HER2/neu expression 0.9% in intrahepatic CC and 8.5% in extrahepatic CC	No treatment data provided	–

In terms of this particular assay’s ability to detect *ERBB2* (HER2/neu) amplification, data were presented by the group from M.D. Anderson Cancer Center at the American Society of Clinical Oncology Conference (ASCO) 2018.^24,25^ Bardelli and colleagues validated the performance of the Guardant360 ctDNA assay retrospectively in a cohort of HER2/neu amplified metastatic colorectal cancer patients treated in the landmark HERACLES study (phase 2 study of lapatinib plus trastuzumab). The group investigated 48 plasma samples from 29 patients. CtDNA was identified in 47–48 samples; ERBB2 amplification was confirmed in 46 of these 47 (97.9%) samples. The authors reported that a threshold of ≥3 copies of ERBB2 in circulation allowed identification of 94% of fluorescence in situ hybridisation-positive patients. This threshold also correlated with clinical response.^24,25^ These results have been validated in other tumor types and cohorts of patients.^25,26^ Liquid biopsy, therefore, appears to be a reliable and valid tool to detect ERBB2 (Her2/neu), which is indeed an actionable finding across many tumor types including cholangiocarcinoma.

HER2/neu blockade has shown favorable results in cancers carrying HER2 aberrations. Javle et al. investigated the response of anti-HER2 therapy in 8 patients with HER2 mutated gallbladder cancer and showed an overall improvement in terms of disease stability (*n* = 3), partial response (*n* = 4), or complete response (*n* = 1) in their entire patient cohort.^27^ In another study, the researchers studied HER2/neu expression in extrahepatic CCA patients (*n* = 84) and reported that anticancer therapy targeting HER2 receptors may be a reasonable option for these patients.^28^ Our patient has responded very well to anti-HER2 drugs and showed an improvement in symptoms, lab results and shrinkage in tumor size. This implicates the use of targeted therapy as a favorable option in CCA alongside other patients who received anti-HER2-based therapy (Table 2).

CCA diagnosis at an advanced stage has very poor outcomes and is often considered incurable. Valle et al.^29^ reported a study of metastatic CCA patients (*n* = 410), and reported median overall survival of 11.7 months and 8.1 months in a cisplatin–gemcitabine group and gemcitabine group, respectively. In the second-line setting, outcomes are extremely poor (progression-free survival, 3–4 months; overall survival, 6 months). Compared to historical reports, outcomes in patients receiving anti-HER2 therapy in HER2/neu+ CCA is by far superior and very encouraging (Table 2).^30,31^

Our patient at one point was given a choice of hospice and was not eligible for any of the trials due to deranged liver function tests. But now on off-label dual-anti-HER2 therapy continues to be on treatment with excellent performance status without any chemotherapy for more than 12 months at present.

For rare, target-rich diseases like CCA, there is a need to consider approval and it is encouraging to see some recent tumor-agnostic approvals, such as immunotherapy for MSI-H and larotrectinib for NTRK-fusion cancers.^9,12^ We believe our case and recent single-arm phase 2 study outcomes warrant consideration toward approval of anti-HER2 agents (trastuzumab/pertuzumab) in patients with CCA with *ERBB2* amplification who have very limited options and poor prognosis.

Furthermore, liquid biopsy testing for HER2 is not routinely performed at most institutions. Our patient’s positive finding would have been missed. In target-rich diseases such as CCA, where tissue acquisition is a challenge, liquid biopsy provides a viable alternative for both diagnostic and monitoring techniques.

In conclusion, we were able to identify *ERBB2* (HER2/neu*)* amplification on liquid biopsy in a patient with CCA and found anti-HER2 therapy as an effective treatment strategy. This will have implications for other patients with CCA in terms of identification of this target and considerations toward anti-HER2 therapy on- or off-trial.

## Methods

The patient provided written informed consent for her case to be presented. Separate IRB/FDA approval not required for off-label use of the anti-HER2 therapy or reporting of the case.

### Reporting summary

Further information on research design is available in the Nature Research Reporting Summary linked to this article.

## Supplementary information

Reporting Summary

## Data Availability

The authors declare that data supporting the findings of this study are available within the paper. Commercially available platforms were employed with results shown in the figures and tables included.

## Supplementary information

**Supplementary information** accompanies the paper on the *npj Precision Oncology* website (10.1038/s41698-019-0091-4).
